# Testicular regression syndrome in children: a retrospective analysis of diagnostic and laparoscopic findings

**DOI:** 10.3389/fped.2026.1755405

**Published:** 2026-02-10

**Authors:** Thuy Nguyen Thi Mai, Khang Do Van

**Affiliations:** 1 Department of Urology, Vietnam National Children's Hospital, Hanoi, Vietnam; 2 Department of Surgery, Hanoi Medical University, Hanoi, Vietnam

**Keywords:** inguinal exploration, laparoscopy, nubbin, testicular regression syndrome, undescended testis

## Abstract

**Purpose:**

Testicular regression syndrome (TRS) is a cause of nonpalpable testis resulting from prenatal testicular involution. This study aims to describe clinical, ultrasonographic, and laparoscopic characteristics and surgical management of TRS, and to evaluate the necessity of inguinal exploration following laparoscopy.

**Materials and methods:**

A retrospective descriptive study was conducted on boys <16 years who underwent laparoscopic exploration for unilateral intra-abdominal nonpalpable testes at Vietnam National Children's Hospital between January 2021 and October 2025 and were diagnosed intraoperatively with TRS. Data collected included age, laterality, ultrasonographic findings, laparoscopic features, the decision to perform inguinal exploration, and histopathology of nubbin. Contralateral testicular size was measured by ultrasonography and compared with age-matched normal values, with *p* < 0.05 considered statistically significant.

**Results:**

Forty-three patients were included; median age at surgery was 26 months (range 9–156 months), and 32 cases (74.4%) involved the left side. Ultrasonography yielded false-positive findings in 12 cases (27.9%). Contralateral testicular volume was larger than normal in children <24 months (0.86 ± 0.23 vs. 0.44 ± 0.14 mL; *p* < 0.001) and 24–60 months (0.90 ± 0.44 vs. 0.57 ± 0.16 mL; *p* = 0.025), but not in those >60 months (*p* = 0.263). Spermatic vessels and vas deferens terminated proximal to the ring in 3 cases (7.0%) and traversed the ring in 40 cases (93.0%). Inguinal exploration was performed in 17/43 patients (39.5%), revealing and excising 13 testicular remnants (30.2%). Specimens sent for histopathology showed no testicular or seminiferous tissue.

**Conclusions:**

Testicular regression syndrome (TRS) is characterized by left-sided predominance and age-dependent compensatory hypertrophy of the contralateral testis. Ultrasonography is unreliable for diagnosis. While inguinal exploration frequently identifies fibrous remnants containing histological markers of regression, the clinical necessity of routine excision remains debated. We advocate for an individualized management approach that balances the benefits of definitive diagnosis against the low risk of malignancy.

## Introduction

Testicular regression syndrome (TRS) is a distinct form of undescended testis in which the testis initially develops but subsequently undergoes complete or near-complete involution, leaving only small fibrous remnants (nibbins) along the course of the spermatic cord. Clinically, affected boys typically present with an underdeveloped hemiscrotum and a nonpalpable testis, while the contralateral testis often shows compensatory hypertrophy (1). Although TRS accounts for a modest proportion of nonpalpable testes, it raises important questions regarding diagnosis, surgical decision-making, and long-term follow-up, particularly because of the very low but inherent risk of malignancy within testicular remnant (2).

**Table 1:** Baseline characteristics and comparison of contralateral testicular volume.

Characteristics	Total Cohort (*N* = 43)
Age at surgery (months)	
Median (IQR)	26 (14–42)
Range	9–156
Laterality, *n* (%)	
Left	32 (74.4%)
Right	11 (25.6%)
Ultrasonography findings	
Correct diagnosis (non-visualized)	31 (72.1%)
False positive (testis-like structure)	12 (27.9%)
Contralateral testicular volume analysis	
Group 1: < 24 months (*n* = 19)	
Study mean ± SD (range)	0.86 ± 0.23 (0.52–1.45)
Normative value^a^	0.44 ± 0.14
*p*-value	<0.001
Group 2: 24–60 months (*n* = 13)	
Study mean ± SD (range)	0.90 ± 0.44 (0.60–1.85)
Normative value^a^	0.57 ± 0.16
*p*-value	0.025
Group 3: > 60 months (*n* = 11)	
Study mean ± SD (range)	1.10 ± 0.77 (0.75–2.90)
Normative value^a^	1.26 ± 0.57
*p*-value	0.263

SD, standard deviation; IQR, interquartile range. Missing data: Volume measurements were unavailable for 5 patients due to incomplete ultrasound records. These were excluded from the volume analysis.

^a^ Normative reference values derived from Liu et al. (6).

**Table 2:** Laparoscopic findings and surgical management stratified by vessel termination.

Variable	Total (*n* = 43)	Intra-abdominal TRS (*n* = 3)	Extra-abdominal TRS (*n* = 40)
Internal inguinal ring status			
Closed, *n* (%)	43 (100%)	3 (100%)	40 (100%)
Open, *n* (%)	0 (0%)	0 (0%)	0 (0%)
Spermatic vessel termination			
Proximal to internal ring	3 (7.0%)	3	0
Traversing internal ring	40 (93.0%)	0	40
Surgical management			
Laparoscopy only (No exploration)	26 (60.5%)	3 (100%)	23 (57.5%)
Inguinal exploration	17 (39.5%)	0 (0%)	17 (42.5%)
Exploration findings (*n* = 17)			
Fibrous nubbin identified & excised	13	–	13
Empty canal/No remnant found	4	–	4

Scrotal ultrasonography is commonly employed as the initial imaging investigation for nonpalpable testes but it has limited diagnostic accuracy in cases of TRS, with frequent misinterpretation of testicular remnants as lymph nodes or other soft-tissue structures (3). Laparoscopy remains the gold standard for both diagnosis and management planning, enabling direct visualization of the spermatic vessels, vas deferens, and their terminal course. However, even after diagnostic laparoscopy, the subsequent management strategy remains a subject of ongoing debate. In cases where the spermatic vessels and vas deferens are seen entering a closed internal inguinal ring, the need for inguinal exploration to identify and excise potential remnants is controversial. Some authors consider laparoscopy alone sufficient, whereas others advocate routine inguinal exploration to eliminate the possibility of the testicular remnant, given its theoretical malignant potential. Reports of histopathological findings in testicular remnants also vary considerably, particularly regarding the presence of seminiferous tubules or germ cells (2, 4, 5).

To provide updated local experience on the diagnosis and management of TRS, with emphasis on the role of inguinal exploration following laparoscopy, we conducted a retrospective study of Vietnamese patients who underwent laparoscopic surgery for TRS at a tertiary pediatric urology center between 2021 and 2025.

## Materials and methods

### Study population

The study included pediatric patients diagnosed with unilateral intra-abdominal undescended testis who underwent laparoscopic surgery at Vietnam National Children's Hospital in Hanoi, Vietnam from January 2021 to October 2025. At our institution, diagnostic laparoscopy is performed routinely as the initial standard procedure for all nonpalpable testes, regardless of preoperative ultrasound findings, to definitively confirm anatomy and avoid negative inguinal explorations due to false-positive imaging.

### Inclusion criteria

Male patients younger than 16 years with unilateral intra-abdominal nonpalpable testis managed laparoscopically, with intra-operative confirmation of absent testis or a severely regressed testis represented only by fibrous remnants (nubbin) on the affected side were included in the study.

### Exclusion criteria

Patients with a history of prior surgery for undescended testis, or patients with congenital syndromes or disorders affecting genital development, including Prader-Willi syndrome, Klinefelter syndrome, persistent Müllerian duct syndrome, or other disorders of sex development were excluded from the study.

### Study design and methods

This was a retrospective descriptive study. A convenient, consecutive sampling approach was applied, including all eligible patients who fulfilled the inclusion and exclusion criteria during the study period.

Data were collected from medical records and included age at surgery, laterality of the affected testis, preoperative ultrasonography findings, intraoperative observations and pathological analysis of the nubbins. During the study period, there was no institutional protocol requiring pathological evaluation for all excised nubbins, thus submission to pathology was at the discretion of the operating surgeon. Volume of the contralateral testes were measured with preoperative ultrasonography. Testicular volume was calculated using the Lambert formula: V = L × W × H × 0.71 (mL), where *L* is length (cm), *W* is width (cm), and *H* is height (cm). These values were compared against age-matched normative reference values for healthy children derived from the study by Liu et al. (6).

For this analysis, patients were categorized into two groups based on the intraoperative laparoscopic anatomy of the spermatic vessels and vas deferens.

#### Intra-abdominal TRS

Spermatic vessels and vas deferens terminated blindly proximal to a closed internal inguinal ring.

#### Extra-abdominal TRS

Spermatic vessels and vas deferens traversed the internal inguinal ring into the inguinal canal.

This categorization is based solely on gross intraoperative findings and the experiences at our center.

### Data processing and analysis

Statistical analysis was performed using IBM SPSS Statistics for Windows, version 20.0 (IBM Corp., Armonk, N.Y., USA). Continuous variables were assessed for normality using the Shapiro–Wilk test. Normally distributed data were presented as mean ± standard deviation (SD), while non-normally distributed data were reported as median and interquartile range (IQR). Categorical variables were summarized as frequencies and percentages. To evaluate compensatory hypertrophy, the mean volume of the contralateral testis was compared with established age-matched normative values using the one-sample t-test. A *p*-value < 0.05 was considered statistically significant.

### Ethical considerations

The study was retrospective and descriptive in nature thus ethical approval was exempt from the institutional review board of Vietnam National Children's Hospital. All patients were anonymized prior to data entry to ensure confidentiality and protect privacy.

## Results

During the study period, 43 patients met the inclusion criteria. The median age at surgery was 26 months, ranging from 9 to 156 months. Left-sided involvement was more frequent, observed in 32 patients (74.4%), while 11 patients (25.6%) had right-sided absence of the testis (Table 1).

All patients presented with an underdeveloped hemiscrotum and a nonpalpable testis on the affected side. Preoperative scrotal ultrasonography produced 12 false-positive findings (27.9%).

Contralateral testicular volume measured on ultrasonography tended to be larger than age-matched normative values, particularly among children aged ≤ 5 years.

In children younger than 24 months, contralateral testis volume was significantly larger than age-matched normal values (*p* < 0.001). A similar pattern was observed in the 24 to 60-month group (*p* = 0.025) (Table 1). In contrast, no significant difference was seen in children older than 60 months (*p* = 0.263), suggesting that compensatory hypertrophy is more pronounced in younger patients.

In all patients (100%), laparoscopy revealed a closed deep inguinal ring. Three patients (7.0%) had the spermatic vessels and vas deferens terminating proximal to the deep inguinal ring, while in the remaining 40 patients, these structures were seen passing beyond the closed ring. Inguinal exploration was performed in 17 cases, among which 13 testicular nubbins were identified and excised (Table 2).

Of the 13 excised remnants, 5 specimens were submitted for histopathological analysis. The remaining 8 nubbins were deemed characteristics for benign regression (with a firm, fibrous appearance with white-tan or yellow discoloration) and were not submitted for pathological examination (Table 3). Pathological examination all revealed fibrous and connective tissue. Notably, dystrophic calcification and hemosiderin deposits, histological markers consistent with regression following a vascular accident, were identified in 3 of the 5 specimens (60%). No seminiferous tubules or viable germ cells were detected in any of the examined cases.

## Discussion

Our cohort had a median age at surgery of 26 months, which is comparable to findings from several recently published larger studies. Mao et al., in a series of 368 children with testicular regression syndrome (TRS), reported a median operative age of 27 months (4). Similarly, Gao et al. analyzed 332 boys with TRS and found a median age of 25 months (2). Although our result (26 months) aligns closely with these real-world data (25–27 months), it remains noticeably later than current guideline recommendations. Major pediatric urological associations, including the American Urological Association (AUA) and the European Association of Urology (EAU), uniformly advise that orchiopexy for cryptorchidism should be performed between 6 and 12 months of age and not later than 18 months (7). This gap between clinical practice and guidelines also appears in other studies. For example, He et al. evaluated 570 children with TRS and reported a mean operative age as high as 38 months, with a very broad range (5–193 months) (1). These findings collectively highlight that, in real-world settings, a substantial proportion of children diagnosed with TRS continue to undergo surgical intervention later than the ideal timeframe before 18 months of age.

One notable finding in our study is the clear predominance of TRS on the left testes. We observed that 32 of 43 cases (74.4%) occurred on the left side, compared with only 11 cases (25.6%) on the right. This is not an isolated pattern unique to our cohort but a well-documented feature consistently reported in the literature. He et al., in a series of 570 patients with TRS, reported 457 left-sided cases (80.2%) and only 113 right-sided cases (19.8%) (1). Similarly, Mao et al. observed a left-sided predominance of 72.6% (267/368) (4). A systematic review by Shepard et al. further corroborated this finding, noting that left-sided involvement typically ranges from 60% to 72.5% (8). The anatomical and developmental basis for this asymmetry has been attributed to differences in the timing and mechanics of testicular descent. The left testis is believed to descend later and possesses a longer spermatic cord, potentially making it more susceptible to intrauterine vascular events such as torsion or ischemia. Taken together, the strong concordance between our findings and those from large international cohorts suggests that our study population is highly representative of the broader TRS spectrum. It also implies that the underlying pathophysiology in these patients likely converges on a shared mechanism, most plausibly an intrauterine vascular accident, a well-recognized etiology with a left-sided predilection.

We identified 12 cases of false-positive ultrasound findings, accounting for 27.9% of the case. This means that in nearly one-third of TRS cases, ultrasonography incorrectly suggested the presence of a testis-like structure. Our results reinforce the existing literature regarding the inherent limitations of ultrasonography in evaluating nonpalpable testes. A systematic meta-analysis by Tasian and Copp demonstrated that ultrasound has a sensitivity of only 45% and a specificity of 78% (9). Due to this lack of reliability, clinical practice guidelines from both the American Urological Association (AUA) and the European Association of Urology (EAU) expressly recommend against the routine use of ultrasonography for diagnosing or localizing nonpalpable testes (10, 11). The main concern is that ultrasound cannot reliably distinguish between a nubbin, an inguinal lymph node, or other soft-tissue structures within the inguinal canal. Such misinterpretation may mislead both surgeons and families and carries the risk of inaccurate operative planning. Our results emphasizes that ultrasonography is not a dependable tool for determining the presence, location, or nature of a nonpalpable testis. Instead, diagnostic laparoscopy remains the gold-standard approach, allowing both definitive diagnosis and appropriate management.

We also observed compensatory hypertrophy of the contralateral testis, reflected by a significantly larger contralateral testicular volume compared with age-matched normative values. This difference was particularly pronounced and statistically significant in children younger than 24 months (*p* < 0.001) and those aged 24–60 months (*p* = 0.025). Notably, however, no significant difference was observed in children older than 60 months (*p* = 0.263). Our findings are consistent with the international literature, which strongly supports the diagnostic value of contralateral testicular hypertrophy as a clinical indicator and a useful predictor of TRS. A recent systematic review by Fateme Tahmasbi et al., synthesizing data from 17 studies, reaffirmed the predictive utility of contralateral testicular hypertrophy (12). Nevertheless, congenital testicular hypertrophy appears to happen within a specific “time window.” The absence of a significant difference in the > 60-month group in our study provides further evidence for this concept. This observation aligns well with prior reports. An 18-year retrospective analysis by Wei et al. showed that the predictive accuracy of contralateral testicular hypertrophy was highest in children aged 0–2 years (73.1%) and declined progressively with increasing age (13). Similarly, Son et al. emphasized that the 6–18-month period is the ideal window for evaluating congenital testicular hypertrophy, as it excludes potential confounding effects from the mini-puberty phase (14).

In this study, we classified patients into two main groups. Group 1 (7.0%; 3/43) consisted of cases in which the spermatic vessels and vas deferens terminated proximal to the closed internal ring, corresponding to intra-abdominal TRS. Group 2 (93.0%; 40/43) included cases in which these structures were seen passing beyond the closed ring into the inguinal canal, consistent with extra-abdominal TRS (Table 2). These data suggest that the vast majority of TRS cases in our cohort likely resulted from a late vascular event (late gestational or perinatal), most plausibly torsion, occurring after the testicular vessels and vas deferens had already traversed the internal ring.

This finding is directly related to an ongoing controversy in the literature: the necessity of inguinal exploration following diagnostic laparoscopy in cases of testicular regression syndrome. Some authors argue that once laparoscopy confirms spermatic vessels and the vas deferens terminate before a closed internal ring, this constitutes sufficient evidence to diagnose testicular regression or agenesis, and further inguinal exploration is unnecessary. This viewpoint has been described in studies by Ismail et al. and Salah & Ahmed (15, 16). However, laparoscopic assessment is not universally reliable. Multiple studies have emphasized that laparoscopy may provide only indirect evidence. Notably, reports by Sturm et al. and Mao et al. demonstrated that inguinal or inguinoscrotal exploration revealed testicular nubbins in 57.8% to 72% of cases, even when laparoscopy initially showed terminating vessels within the abdomen (5, 17). Thus, no absolute consensus currently exists. Some surgeons elect to stop when laparoscopy shows intra-abdominal termination of spermatic cord and no residual testicular tissue is suspected, whereas others continue to advocate routine inguinal exploration to definitively exclude the presence of a nubbin (Table 3).

**Table 3:** Comparison of histopathological findings and management recommendations across studies.

Study	Sample size (*n*)	Seminiferous tubules (%)	Viable germ cells (%)	Other histological findings	Recommendation for remnant excision
Current study	5^a^	0%	0%	Fibrosis, hemosiderin, calcification (60%)	Individualized/Consider
Mao et al. (4)	368	7.1%	4.5%	Fibrosis, dystrophic calcification	Yes (Routine)
He et al. (1)	513^b^	6.6%	3.1%	Calcification (41%), Hemosiderin (11%)	Controversial/Selective
Gao et al. (2)	332	6.3%	2.1%	Fibrosis, calcification	May not be necessary
Nataraja et al. (19)^c^	1,455	10.7%	5.3%	Fibrosis, calcification, hemosiderin	Yes (Routine)
Sturm et al. (5)	207	5.3%	1.0%	Fibrosis, calcification	May not be necessary
Bader et al. (20)	208	15.0%	11.0%	Not detailed	Yes (Routine)

^a^ *n* for Current Study refers to the number of specimens submitted for histopathological examination.

^b^ He et al. reported on 513 excised remnants out of 570 cases.

^c^ Nataraja et al. is a systematic review aggregating data from 29 studies.

The question of whether testicular remnants should be excised remains a subject of ongoing debate. Some authors argue that the malignant potential of these remnants is extremely low, as most contain no viable testicular tissue. Heksch et al. emphasized the absence of consensus within the medical community, and Sturm et al. reported no viable germ cells in any of the 26 remnants examined, thereby questioning the true benefit of surgical excision (5, 18). On the other hand, Mao et al. noted that although uncommon, some remnants may still contain seminiferous tubules (approximately 5%–10%) or germ cells (< 2%), and cases of intratubular germ cell neoplasia have been documented (17).

A notable limitation of our study was the selective submission of specimens for histopathology, with only 5 of the 13 excised remnants (38.5%) analyzed microscopically. This pattern of selective evaluation reflects the specific clinical and socio-economic context of our practice during the study period (2021–2025) and can be attributed to three primary factors.

First, clinical decision-making was heavily influenced by macroscopic assessment. In the majority of cases, the excised nubbins exhibited distinctive gross features of benign regression, often leading to the omission of further pathological examination.

Second, given that the theoretical risk of malignancy in testicular remnants in children under 3 years of age is extremely low, some surgeons viewed routine pathological analysis as an added financial burden with limited immediate clinical benefit, and thus reserved it for cases with atypical findings. Third, there was a lack of a standardized protocol at our institution during the early phase of the study. The decision to submit tissue was largely left to the individual surgeon's discretion, resulting in variability in practice.

However, recognizing the potential, albeit rare, risk of malignancy or the presence of viable germ cells as reported in the literature (17), we acknowledge that macroscopic inspection alone is insufficient to definitively exclude pathology. In the 5 specimens that were analyzed, we identified fibrous tissue with dystrophic calcification and hemosiderin deposits in 3 cases (60%). These histological markers serve as a footprint of a past vascular accident. Consequently, to address these limitations and eliminate diagnostic uncertainty, we have since updated our institutional workflow to mandate histopathological examination for all excised nubbins, regardless of their gross appearance.

This study provides one of the first detailed analyses of TRS in Vietnamese children, offering region-specific insights into diagnosis and intraoperative management that complement existing data from Western and Chinese cohorts. However, as our cohort is small and from a single center, it is difficult to infer guidance and recommendation from these experiences. Future large-scale studies will be essential to refine treatment recommendations, particularly regarding surgical timing and the role of inguinal exploration.

## Conclusion

Our study confirms that Testicular Regression Syndrome (TRS) is characterized by a marked left-sided predominance and is frequently accompanied by compensatory hypertrophy of the contralateral testis, particularly in infants and young children. We demonstrated that preoperative ultrasonography is unreliable due to substantial false-positive rates.

Regarding the controversy of surgical management, our institutional experience suggests that finding spermatic vessels traversing the internal inguinal ring warrants consideration for inguinal exploration, as this frequently leads to the identification of fibrous remnants. However, given the low risk of malignancy and the observational nature of our data, we advocate for an individualized approach to remnant excision, balancing the benefits of definitive histological diagnosis against the extent of surgical intervention.

## Data Availability

The raw data supporting the conclusions of this article will be made available by the authors, without undue reservation.
